# Convert Harm into Benefit: The Role of the Al_10_CaFe_2_ Phase in Al-Ca Wrought Aluminum Alloys Having High Compatibility with Fe

**DOI:** 10.3390/ma16237488

**Published:** 2023-12-02

**Authors:** Tianying Shen, Shasha Zhang, Zili Liu, Shuaipeng Yu, Junchao Jiang, Xuewei Tao, Torgom Akopyan, Nikolay Belov, Zhengjun Yao

**Affiliations:** 1 College of Materials Science and Technology, Nanjing University of Aeronautics and Astronautics, Nanjing 210016, China; sx2106089@nuaa.edu.cn (T.S.);; 2 Key Laboratory of Materials Preparation and Protection for Harsh Environment, Ministry of Industry and Information Technology, Nanjing 210016, China; 3 School of Materials Science and Engineering, Nanjing Institute of Technology, Nanjing 211167, China; 4 Department of Metal Forming, National University of Science and Technology MISIS, 4 Leninsky pr, Moscow 119049, Russia

**Keywords:** wrought aluminum alloys, ternary Al-Ca-Fe phase, high-Fe compatibility, microstructure evolution, crystal structure

## Abstract

The compatibility of the wrought Al-Ca alloy with the element Fe was investigated in the present study. In this work, both the Al-Ca alloy and Al-Ca-Fe alloy were synthesized through melting, casting, heat treatment, and rolling. A new ternary Al-Ca-Fe eutectic phase, identified as Al_10_CaFe_2_ with an orthorhombic structure, demonstrated enhanced performance, as revealed by nanoindentation tests. Combining the results of the nanoindentation and EBSD, it can be inferred that during the rolling and heat treatment process, the divorced eutectic phases were broken and spheroidized, and the structure of the Fe-rich alloy became finer, which promotes the formation of fine grains during the process of dynamic recrystallization and effectively hindered the grain growth during thermal treatment. Consequently, the strength of the as-rolled Al-Ca alloy was improved with the addition of 1 wt.% Fe while the ductility of the alloy was maintained. Therefore, adding Ca into the high-Fe content recycled aluminum altered the form of the Fe-containing phases in the alloy, effectively expanding the application scope of recycled aluminum alloy manufacturing. This approach also offered a method for strengthening the Al-Ca aluminum alloys. Compared to the traditional approach of reducing Fe content in alloys through metallurgical means, this study opened a new avenue for designing novel, renewable aluminum alloys highly compatible with impurity iron in scrap.

## 1. Introduction

The concepts of energy conservation and emission reduction are increasingly recognized as critical issues in the aluminum manufacturing industry [1,2,3,4,5]. Given its infinite recyclability, aluminum has great recycling value. This is evidenced by the substantial energy savings achieved in recycling and remanufacturing scrap aluminum, as opposed to primary aluminum smelting. The energy required to melt aluminum from the waste is only about 5% of the energy needed for extracting the primary aluminum from the ore [6]. Not only that, the replacement of primary aluminum by secondary aluminum can also reduce greenhouse gas emissions. The emission of primary aluminum extraction from ore is about 12 kg·CO_2_/kg, while the emission of secondary aluminum recovered from waste is only about 0.6 kg·CO_2_/kg [7]. Consequently, recycling scrap aluminum could align the energy and carbon balance of aluminum manufacturing more closely with the needs of the green economy and sustainable development [8]. 

However, the process of recycling and remanufacturing aluminum alloys from scrap is fraught with numerous challenges. The most serious problem is that the waste aluminum recycling process will inevitably bring in some harmful elements or make the content of some elements exceed the limit. In wrought aluminum alloys, Fe normally acted as a harmful element [9,10,11,12], forming coarse brittle phases such as Al_3_Fe, Al_6_(Mn, Fe), Al_5_FeSi, etc., resulting in a decline in alloy properties [13,14,15]. Therefore, the properties of the alloy with recycled aluminum are often lower than those with primary aluminum, and it is usually used on some occasions with low-performance requirements. In the process of recycling aluminum alloys, Fe is more difficult than other elements, which is manifested in two aspects. On the one hand, Fe is difficult to remove by metallurgical means in the process of smelting and refining; on the other hand, Fe tends to accumulate progressively in the recycling of secondary aluminum alloy [16,17,18]. These reasons ultimately lead to Fe in the recycled aluminum alloy being much higher than the primary aluminum alloy. To solve this problem, a small amount of Mn, Cr, K, or other elements is often added to reduce the harm of Fe [19,20,21,22]. However, this method is only suitable for low Fe content in the process of industrial manufacturing aluminum alloy, and the addition of new elements often implies a more complex production process [21]. Design of new alloy systems that are compatible with the high impurities in the aluminum scraps is in urgent need [6,23]. Beyond the conventional aluminum alloy series, the Al-Ca alloys are found to have good castability and mechanical forming ability with unique properties [24,25,26,27,28]. Ca has a low density, and it is abundantly available. During the solidification process of Al-Ca alloy, a eutectic reaction of L—(Al) + Al_4_Ca occurs at 617 °C with a Ca content of 7.6% [29]. When the Ca content is excessively high, it leads to the formation of coarse primary Al_4_Ca phases. Such an Al-Ca eutectic alloy exhibits a refined microstructure and excellent deformation processing properties [30]. The eutectic Al_4_Ca phase appears as fine lamellae, exerting a beneficial influence on the performance of the alloy [31]. Furthermore, at elevated temperatures, the network distribution of Al_4_Ca can effectively strengthen the grain boundaries, thereby inhibiting grain growth [28]. Fe exists in the form of a ternary phase in Al-Ca alloy, inhibiting the alloy from forming a coarse primary brittle phase like Al_3_Fe [32]. However, the information on microstructure evolution and crystal structure of this new phase has not been studied. 

To provide a research basis for solving the harm caused by the high-Fe content of recycled aluminum alloys, more Fe (1%) was added than ordinary wrought aluminum alloy. The Al-Ca alloy and Al-Ca-Fe alloy were synthesized through melting, casting, heat treatment, and rolling, and the microstructure, mechanical properties, microstructure evolution, and strengthening mechanism of the alloys were studied in this work.

## 2. Experimental

### 2.1. Materials

**Figure 1:**
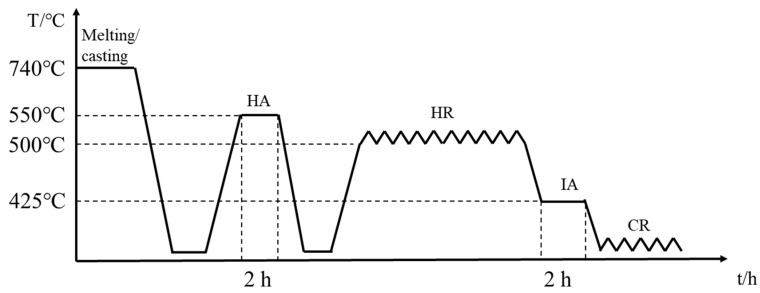
Scheme of obtaining experimental samples.

The compositions of the Al-Ca and Al-Ca-Fe systems were determined according to the chemical analysis performed by ICP-OES (Agilent 720ES, Agilent Technologies, Santa Clara, CA, USA). The Al-Ca alloy containing 3.63%Ca, 0.17%Fe and Al-Ca-Fe alloy containing 3.63%Ca, 1.03%Fe were synthesized, and Fe (10%Fe and 90%Al reagent) and Ca (10%Ca and 90%Al reagent) were added via master alloys (all contents given in wt.%). The melting process of the alloy was carried out in a resistance furnace in an air environment. After the refining process, the two alloys were held between 720 °C and 740 °C for 30 min and then cast into a cylindrical copper mold (140 mm in width, 80 mm in height, and 12 mm in thickness). After homogenizing annealing (HA) at 550 °C for 2 h, the alloys were hot rolled (HR) on a laboratory two-roll mill from 12 mm to 2.4 mm. During the hot rolling process, the furnace temperature was maintained at 500 °C. After the sample was held for 20 min, the first rolling was performed with a reduction of approximately 0.5 mm. After two consecutive rolling passes, the sample was reheated and held in the furnace for 5 min before being taken out for the next rolling pass. Intermediate annealing (IA) was carried out at 425 °C for 2 h, and then the alloys were cold rolled (CR) from 2.4 mm to 1.2 mm. The reduction in each cold rolling pass was approximately 0.5 mm. The scheme of the experiments and the main stages of the processing route are summarized in Figure 1.

### 2.2. Methods

Tensile tests were carried out using a CMT 5205 universal testing machine (MTS, Shenzhen, China) with a scale distance of 20 mm and a drawing rate of 1 mm/min in the rolling direction. The nanoindentation test was performed on the Agilent G200 device (Agilent Technologies, Santa Clara, CA, USA). The indents were obtained by applying a maximum load of 8 mN and the loading, holding, and unloading times were all 10 s. The samples were polished according to standard metallographic procedures, and Keller reagent (HCl 1.5%, HF 1%, HNO_3_ 2.5%, H_2_O 95%) was used to corrode the sample surface. The microstructures of the samples were observed using a ZEISS Imager A2m metallographic microscope (OM) (ZEISS, Oberkochen, Germany). The samples were also characterized using a Malvern Panalytical Empyrean X-ray diffraction (XRD) (Malvern, UK) with Cu Ka radiation at 40 mA and 40 kV (Smartlab 9 KW). The range of diffraction angles (2θ) measured during XRD Diffraction was from 20° to 80°. The measurements were made at a scan speed of 5° per minute. The microstructural analysis was performed on an ionization double beam scanning electron microscope (SEM) type Tescan Lyra3GM (Brno, Czech Republic). A transmission electron microscope (TEM) type FEI Tecnai G20 (Hillsboro, OR, USA) was employed to determine the crystal structure of the phases existing in the alloy. An energy dispersive spectrometer (EDS) was used to analyze the phase spectrum of the samples. A JXA-8530F electron probe micro-analyzer (EPMA) (JEOL, Tokyo, Japan) was used to analyze the elemental distribution mapping of the samples. The electron backscatter diffraction (EBSD) was performed using an EDAX Hikari Plus (Warrendale, PA, USA) on electrolytically polished rolled samples, aiming to obtain statistical data on the grains of the alloy and its stress state.

## 3. Results

### 3.1. Microstructure

**Figure 2:**
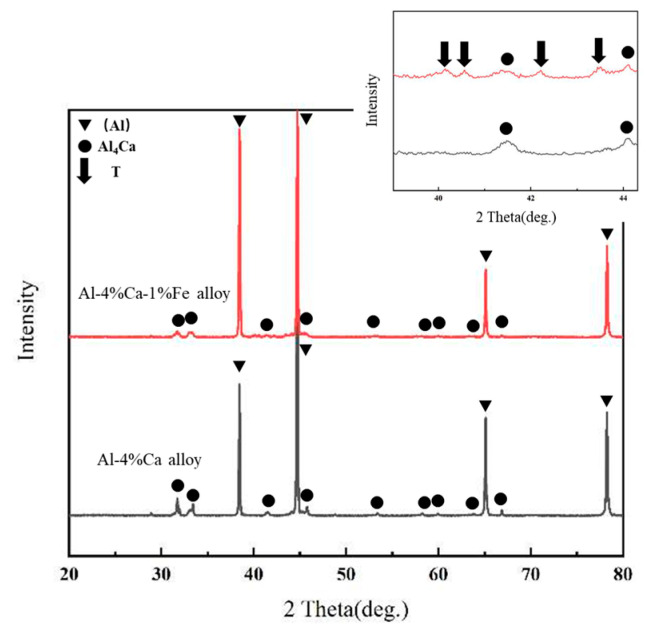
X-ray diffraction of the rolled Al-Ca alloy and Al-Ca-Fe alloy.

The XRD results showed that a new phase was formed, as shown in Figure 2. The α-Al diffraction peaks were detected in both samples. In addition to α-Al, the diffraction peaks of the Al_4_Ca phase are relatively weak but still identifiable. Compared with that of the Al-Ca alloy, some weak diffraction peaks that did not belong to α-Al or Al_4_Ca were also detected in the as-rolled XRD diffraction pattern of the Al-Ca-Fe alloy, which indicated that a small new amount of the Al-Ca-Fe phase is formed in the Al-Ca-Fe alloy, and temporarily labeled as T phase (ternary phase).

**Figure 3:**
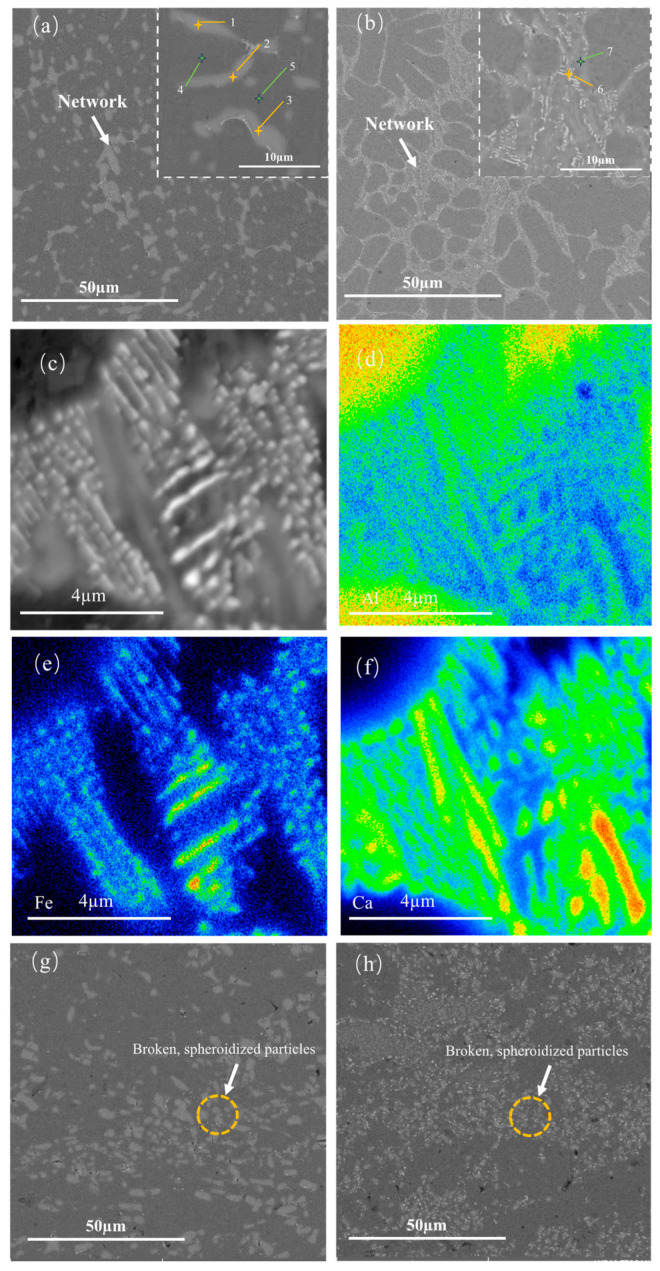
BSE and EMPA images of the experimental alloys (**a**) BSE images of as-cast Al-Ca alloy; (**b**) BSE images of as-cast Al-Ca-Fe alloy; (**c**–**f**) EPMA mapping results of as-cast Al-Ca-Fe alloy, corresponding mapping showing Al (**d**), Fe (**e**) and Ca (**f**) of microstructure (**c**); (**g**) BSE images of as-rolled Al-Ca alloy; (**h**) BSE images of as-rolled Al-Ca-Fe alloy.

Figure 3a,b depict the BSE images of the as-cast Al-Ca alloy and Al-Ca-Fe alloy, with the detailed EDS results presented in Table 1. The element points scanning results in Table 1 reveal that the atomic ratio of Al to Ca in the grey phase approximates 4:1. Combined with the XRD results, the as-cast Al-Ca alloy contains two main phases: the black matrix of primary (Al) and grey divorced eutectic Al_4_Ca which was distributed in a network or semi-network. Compared to the microstructure of the as-cast Al-Ca alloy, the microstructure of the Al-Ca-Fe alloy was finer. Since the casting processes of both alloys were identical, it could be inferred that the addition of Fe element refined the eutectic structure of the alloy. To elucidate the chemical distributions of the eutectic phases in the as-cast Al-Ca-Fe alloy, the EMPA results are depicted in Figure 3c–f. The distribution of three elements distinguished the eutectic structure from (Al). In (Al), there were almost no Fe and Ca elements. An additional ternary phase containing Al, Ca, and Fe elements was found in the as-cast Al-Ca-Fe alloy, which was referred to as the T phase in this paper. The T phase had a thin slatted or spherical shape, which was finer than that of Al_4_Ca. The T phase was symbiotic with the Al_4_Ca, and these two phases formed a laminar morphology. The network structure formed by the T phase and Al_4_Ca is shown in Figure 3b, which was more continuous than that of the as-cast Al-Ca alloy, and no coarse primary phase formation was found. The network structure of the eutectic phase resulted in very fine grains in the as-cast alloy. Figure 3g,h show that the eutectic phases of rolled alloys were fragmented and refined, with the phase in the Al-Ca-Fe alloy being notably finer than that in the Al-Ca alloy.

### 3.2. Mechanical Properties

**Figure 4:**
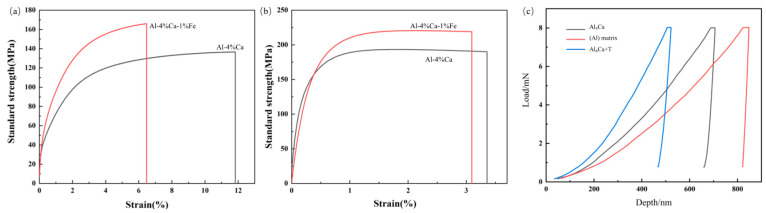
Stress-strain curves of Al-Ca alloy and Al-Ca-Fe alloy (**a**) as cast; (**b**) as rolled; (**c**) nanoindentation load-depth curve of different phases.

As shown in Figure 4a and Table 2, the tensile strength of the as-cast Al-Ca-Fe alloy was about 27 MPa higher than that of the Al-Ca alloy but the elongation was lower. The as-rolled Al-Ca-Fe alloy demonstrated the mechanical properties of 221 MPa in ultimate strength and 188 MPa in yield strength, which was higher than that of the Al-Ca alloy (Figure 4b). The yield strength of the Al-Ca-Fe alloy was about 16 MPa higher than that of the Al-Ca alloy, which was consistent with the calculated results of fine crystal strengthening. The data presented in Table 2 demonstrated that the elongation of the rolled alloys was similar, which meant that the addition of 1 wt.% Fe maintained plasticity while the strength was increased, and the matching of strength and plasticity was achieved.

Figure 4c is the nanoindentation load-depth curve of the alloy, which directly shows the hardness differences of the (Al) matrix, Al_4_Ca, and Al_4_Ca + T phases. The elastic strain to failure (H/E) and the plastic deformation resistance factor (H^3^/E^2^) were calculated based on Young’s modulus (E) and hardness (H), and the results were shown in Table 3, indicating that the combination of Al_4_Ca + T phase was the relative tougher phase [33]. However, the H/E and H^3^/E^2^ differences between Al_4_Ca + T and Al_4_Ca were not large, which also made the alloy maintain good plasticity. The relatively tougher eutectic phase and fine grain made Al-Ca-Fe alloy exhibit better mechanical properties.

Compared to the Al-Mg and Al-Mn alloys, the Al-Ca-Fe alloys have very few solid-solution atoms, and their strength is mainly improved through fine-grain strengthening rather than precipitation strengthening or solid-solution strengthening. This type of alloy can add additional trace elements (such as Sc, Zr) to form a dispersed second phase to improve strength, endowing the alloy with excellent deformation properties as well as electrical and thermal conductivity [26,34]. Such alloys could simplify the heat treatment process, save energy, and reduce carbon emissions.

## 4. Discussion

**Figure 5:**
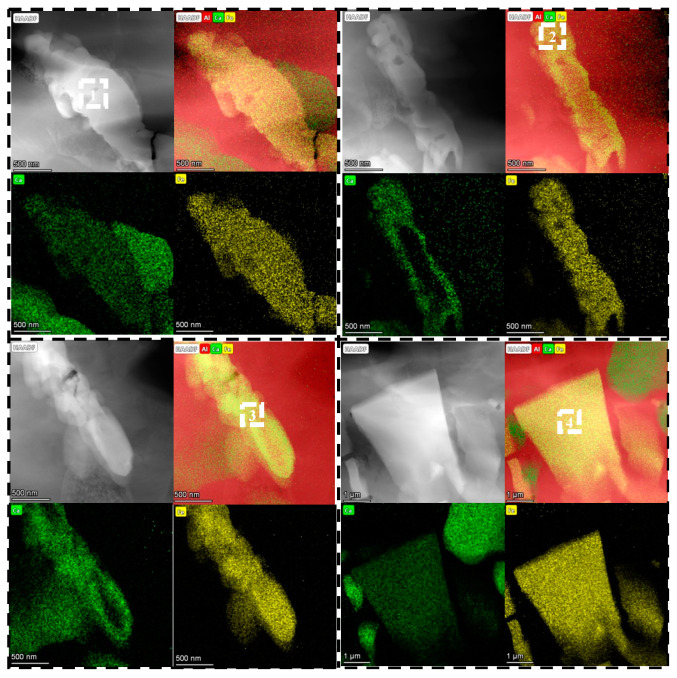
STEM EDS mapping results of four different regions of the as-rolled Al-Ca-Fe alloy with green representing Ca and yellow representing Fe, and the energy spectrums of the selected regions are shown in Table 4.

The STEM EDS mapping results of the as-rolled Al-Ca-Fe alloy are shown in Figure 5, and the results of the chemical composition analysis of the area shown are summarized in Table 4. The EDS mapping allowed for a clear distinction between the T phase and the Al_4_Ca phase. The rolled T phase mainly existed in a rod-like form, but there were also a few block-like structures present. The atomic ratio of Ca and Fe was close to 1:2. The ternary phase of the Al-Ca-Fe in the CIF (Crystallographic Information File) may exist in the form of Al_8_CaFe_4_, and the experimental composition proved that the T phase of Al-Ca-Fe in the alloy was not Al_8_CaFe_4_, but was close to Al_10_CaFe_2_, similar to Al_10_CeFe_2_ and Al_10_LaFe_2_. It was also possible to observe the Al_4_Ca phase near the T phase, which was the green area in the figure.

**Figure 6:**
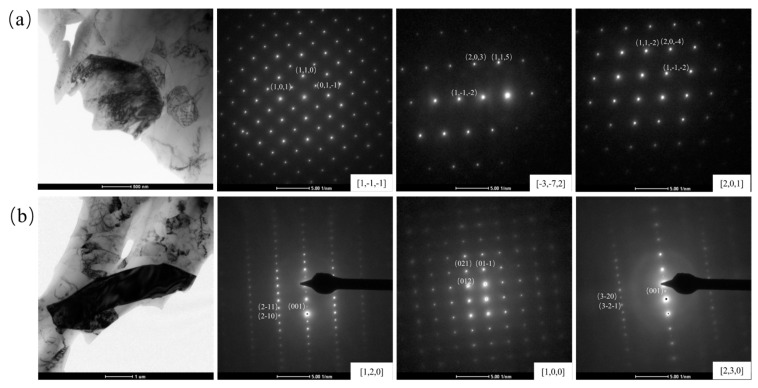
TEM BFI and SADPs taken from the Al_4_Ca phase (**a**) with **B**_1_ = [1, −1, −1], **B**_2_ = [−3, −7,2], and **B**_3_ = [2, 0, 1], TEM BFI and SADPs taken from the T phase (**b**) with **B**_1_ = [1, 2, 0], **B**_2_ = [1, 0, 0] and **B**_3_ = [2, 3, 0].

The crystal structure of the Al_4_Ca and T phase was identified from the TEM observations. The electron diffraction patterns of the unknown phases were taken from three different incident beam directions (**B**) by rotating the sample foil. Based on three different SADPs (Selected Area Diffraction Patterns) in Figure 6, the structure could be determined as tetragonal with the lattice parameters *a* = *b* = 0.42426 nm, *c* = 1.1355 nm, and *α* = *β* = *γ* = 90°, and it was consistent with the CIF of Al_4_Ca [35]. In the same way, the lattice parameter of the T phase was *a* = 0.5000 nm, *b* = 0.5383 nm, and *c* = 0.8718 nm, and it belonged to the orthorhombic structure with *α* = *β* = *γ* = 90°. It was known that the Al_10_CeFe_2_ and Al_10_LaFe_2_ had similar crystal structures and were both orthorhombic structures [36,37]. Therefore, the calculation results showed that the Al_10_CaFe_2_ had a similar orthorhombic structure, which was consistent with the speculation.

Al is face-centered cubic as the CIF shows, and *a* = 0.4049 nm. Therefore, there are obvious differences in structure and lattice parameters between the T phase and Al. In addition, the average length of the T phase was larger than tens of nanometers, and the T phase is a hard phase. Thus, the strengthening by the T phase is the Orowan dislocation bypass mechanism. According to Orowan strengthening theory, the increment of yield strength increased by precipitate can be calculated using the following equation [38]:

(1) $$∆\sigma_{or}=M\frac{0.4Gb}{\pi \sqrt{1-v}}\frac{ln\left(\frac{2r}{b}\right)}{\lambda }$$

where *M* = 3.06 is the Taylor factor, *G* = 25.4 GPa is the shear modulus of Al, *b* = 0.286 nm is the magnitude of the Al Burgers vector, *ν* = 0.35 is the Poisson’s ratio of pure Al matrix, *r* is the mean precipitate radius and *λ* is the average inter-precipitate spacing. So, based on Equation (1), the strengthening effect of the precipitate was determined by the mean precipitate radius and the average inter-precipitate spacing. As shown in Figure 3g,h, the eutectic phases in the as-rolled Al-Ca-Fe alloy were significantly finer compared to the eutectic phases in the Al-Ca alloy. The dispersity of the eutectic phase in both alloys was approximately the same. Therefore, the strengthening effect provided by the second phase in the Al-Ca-Fe alloy was greater than that in the Al-Ca alloy.

**Figure 7:**
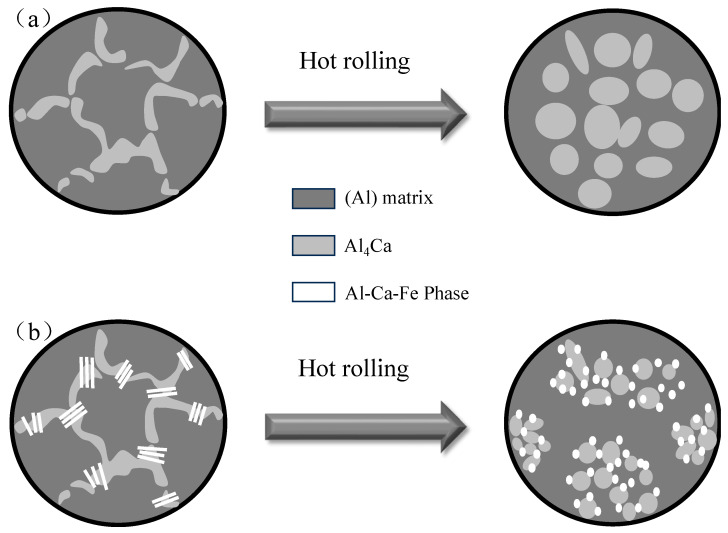
Schematic representation of the eutectic phase spheroidization during the hot rolling process. (**a**) Al-Ca alloy; (**b**) Al-Ca-Fe alloy.

As shown in Figure 7, the T phase exhibited a lamellar morphology in the as-cast Al-Ca-Fe alloy. Considering the nanoindentation results for the Al-Ca-Fe alloy (Table 3), the eutectic structure containing the T phase had enhanced resistance to both elastic and plastic deformation, hindering the deformation of the eutectic structure during the tensile process. This ultimately resulted in higher strength but reduced ductility of the as-cast Al-Ca-Fe alloy (Figure 4a) [28,39]. The spherical shape of the T phase, in comparison to the lamellar shape, reduced the cleavage of the matrix, decreasing stress concentration. Consequently, the elongation rates of the two alloys in the rolled state were similar (Figure 4b).

Previous studies have observed that the eutectic Al_4_Ca fractured after annealing at 450 °C for 3 h, began to spheroid after annealing at 500 °C for 3 h, and was completely spheroidized after annealing at 600 °C for 3 h. Figure 3c–f shows the microstructure of the as-cast Al-Ca-Fe alloy, featuring Al_4_Ca and the T phase in a lamellar morphology. With a constant volume of the eutectic phase, its spherical morphology demonstrated the lowest surface energy. Therefore, the transformation of the Al_4_Ca and the T phase from lamellar to spherical morphology leads to a reduction in the system’s free energy, indicating a spontaneous process. According to the Gibbs–Thompson effect [40], there was a curvature difference between the ends and the center of the lamellar structure. As a result, the solute flowed from the center to the ends, causing thinning in the center and widening at the ends [41]. After the lamellar phase was transformed into a rod-like shape, the perturbation mechanism induced by Rayleigh’s capillarity would take effect [42]. Additionally, since the initial morphology of the T phase was thinner compared to that of the Al_4_Ca phase, the spheroidized T phase also exhibited a finer morphology. Finally, spherical eutectic phases were formed. The initial distribution of the Al_4_Ca and the T phase (Figure 3c–f) was also retained after spheroidization (Figure 5), as shown in Figure 7. The microstructure obtained through thermal deformation is completely spheroidized, even at a temperature lower than 500 °C. This was because deformation-induced spheroidization (DIS) [43] reduced the temperature requirement for complete spheroidization, and the crystal defects generated during the deformation process reduced the energy required for spheroidization. Following the rolling of the Al-Ca-Fe alloy, its eutectic phase particles were observed to be finer than those in the Al-Ca alloy. This could be attributed to the T phase acting as a modifier during high-temperature rolling, as depicted in Figure 3h. On the one hand, it provided more non-uniform nucleation points during the spheroidization of the Al_4_Ca phase. This effect was evident as, during the high-temperature hot rolling process, the T phase, being a fine hard phase, existed symbiotically with Al_4_Ca in the ternary eutectic reaction, thereby enhancing the spheroidization of Al_4_Ca. On the other hand, the T phase functioning as a hard particle impeded the growth of the spheroidized particles of the Al_4_Ca phase.

**Figure 8:**
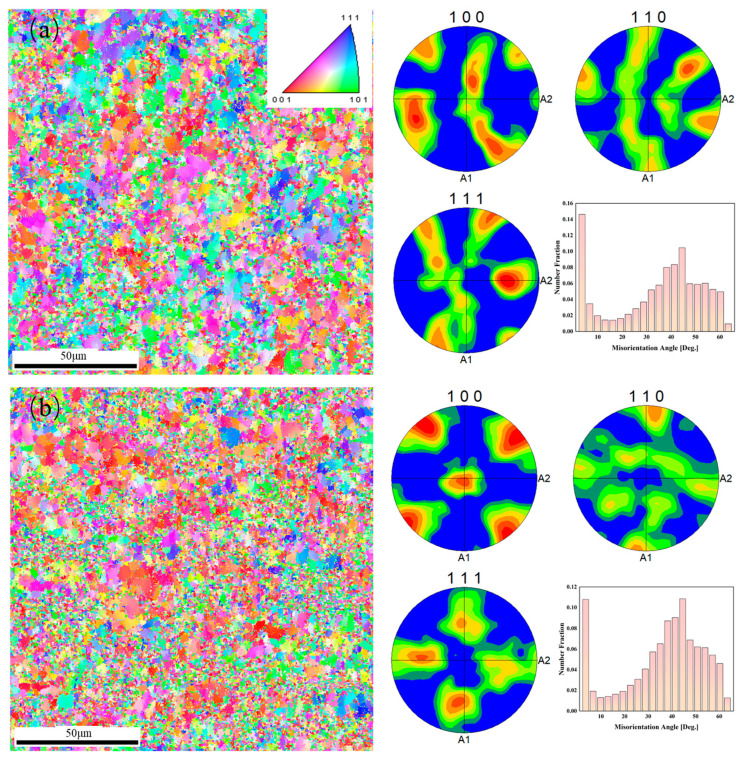
EBSD IPF maps, PF images, and misorientation angle distribution of experimental alloys: (**a**) Al-Ca alloy; (**b**) Al-Ca-Fe alloy. The histograms quantify the distribution of the misorientation angle values, with a shift to a lower misorientation angle for Al-Ca-Fe alloy.

Figure 8 shows the EBSD results of the rolled Al-Ca alloy and Al-Ca-Fe alloy. In Al-Ca alloy, the orientation of α-Al was more distributed in [001] and [111], while in Al-Ca-Fe alloy, the orientation of α-Al was only more distributed in [001]. The PF images showed that the highest density texture of the Al-Ca alloy occurred at {111}, with a maximum density of 4.5; the highest density texture of the Al-Ca-Fe alloy appeared at {100}, and the high-density texture appeared at 45° to the A2 axis (rolling direction), with a maximum density of 3.6. According to the Al-Fe, the Al-Ca binary phase diagram, the maximum solid solubility of Fe in α-Al was 0.04%, while that of Ca was lower, only 0.01% [44,45]. The low solid solubility of the Fe and Ca elements in α-Al especially at high temperatures made them difficult to diffuse in the form of solid solution atoms during hot rolling. The addition of 1 wt.% Fe reduced the small angle grain boundaries which indicated that the as-rolled Al-Ca-Fe alloy had a higher degree of recrystallization during the hot process. 

**Figure 9:**
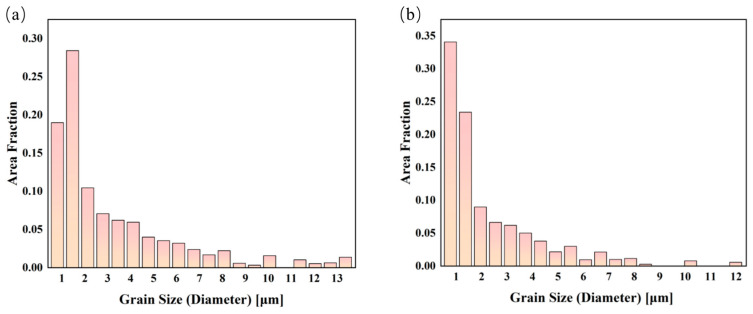
Grain size distribution (total normalized fraction = 1) of experimental alloys: (**a**) Al-Ca alloy; (**b**) Al-Ca-Fe alloy.

From the grain size distribution in Figure 9, it was calculated that the average grain size of the Al-Ca alloy is 3.2 µm, whereas the Al-Ca-Fe alloy exhibits a smaller average grain size of 2.3 µm. It was reported that a large density of dislocations was formed near the intermetallic phase during the rolling process, which created a favorable position for the formation of recrystallization nuclei [19]. Combined with grain size distribution results (Figure 9), it was proved that the addition of Fe promoted the nucleation of recrystallization and hindered the growth of grain, resulting in grain refinement during the high-temperature process. Grain boundary strength can be described by the Hall–Petch relationship:

(2) $$\sigma_{GB}=\sigma_{0}+kd^{-1/2}$$

where *σ*_0_ is the inherent resistance of the lattice to the movement of the dislocation. In general, the σ_0_ value of aluminum alloy is 20 MPa, and *k* is the grain boundary strengthening coefficient, which is 0.17 MN/m^3/2^ [46]. As the average grain diameter (d) of two alloys was obtained, the calculation of yield strength increment caused by the grain size effect is about 17 MPa. Based on the yield strength data of the two alloys in the as-rolled state shown in Table 2, there is a difference of approximately 15 MPa, which is consistent with the calculated results.

**Figure 10:**
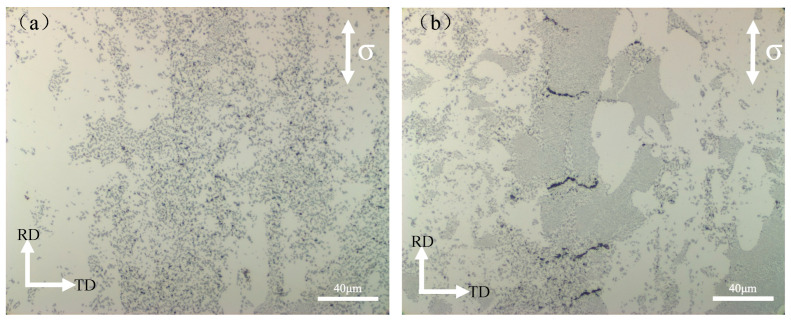
OM images of the tensile fracture outer section of rolled alloys: (**a**) Al-Ca alloy; (**b**) Al-Ca-Fe alloy.

Figure 10 is the OM images of the tensile fracture outer section of the Al-Ca alloy and Al-Ca-Fe alloy as rolled. Comparing the two figures, we found that the Al_4_Ca of the Al-Ca alloy presented a uniformly distributed spherical shape, which was completely different from the as-cast structure. The network structure completely disappeared, and the pores were dispersed in the matrix of the Al_4_Ca phase dense area. The eutectic Al_4_Ca + T phase of the Al-Ca-Fe alloy was finer, the network-like fibrous structure was retained along the rolling direction, and the transverse network structure disappeared. The pores were concentrated in the matrix of the Al_4_Ca + T phase dense area and formed transverse microcracks after aggregation. Figure 10b showed that the pores aggregated and formed secondary cracks in the Al4Ca + T phase, indicating that the Al_4_Ca + T phase had a higher strength compared to the pure Al_4_Ca and therefore bear the load preferentially, which made the alloy have better mechanical properties. At the same time, it was observed that the smaller the particles, the more obvious the strengthening effect [14]. These fracture behaviors revealed that the concentrated distribution of the Al_4_Ca + T phase and the fine spherical morphology provided additional strength enhancement to the alloy. In summary, the presence of a fine globular phase, small-sized grains surrounding the particles, and a high-volume fraction of the eutectic phase collectively contributed to inhibiting the early formation of pores and hindering crack propagation, ultimately preventing failure.

## 5. Conclusions

In this work, two alloys (Al-Ca and Al-Ca-Fe) were prepared by casting, rolling, and heat treatment. The following conclusions could be drawn:

1. The as-cast structure of the alloy presented submicron grains and network/semi-network eutectic structure. The results of the SEM and EPMA showed that the microstructure of the alloy was composed of (Al)+ eutectic phase. After adding 1%wt. Fe, a ternary phase of Al, Ca, and Fe (T phase) with the composition of the Al_10_CaFe_2_ was formed. The lattice parameter of the T phase was *a* = 0.5000 nm, *b* = 0.5383 nm, and *c* = 0.8718 nm, and it belonged to the orthorhombic structure with *α* = *β* = *γ* = 90°.
2. The rolled Al-Ca-Fe alloy had about 31.5% higher tensile strength and 64.5% higher yield strength than the cast alloy. The tensile strength of the Al-Ca-Fe alloy in the rolled state was 27 MPa higher than that of the Al-Ca alloy, and the elongation was maintained, which indicates that the addition of Fe was beneficial to Al-Ca deformed aluminum alloy. The nanoindentation data of the (Al) matrix, Al_4_Ca, and Al_4_Ca + T phase was compared, where the H/E and H^3^/E^2^ of the Al_4_Ca + T phase was the highest, contributing to the strength of the alloy.
3. The Al-Ca-Fe alloy had better mechanical properties than the Al-Ca alloy, which was attributed to the refinement of the spheroidized eutectic structure at high temperature by the T phase and the refinement of the grains during recrystallization. The fine spherical phase, the small grains around the particles, and the eutectic phase with a high-volume fraction all help to prevent the early formation of pores or the propagation of cracks, thereby preventing failure.

In summary, the Al-Ca-Fe alloy demonstrated compatibility with high-Fe content, simultaneously exhibiting effectively improved mechanical properties. This research provides a new direction in addressing the issue of high-Fe content in recycled aluminum alloys through the design of innovative Al-Ca-based wrought aluminum alloys.

## Figures and Tables

**Table 1:** Concentrations of the alloy composition in the spectrum (at%).

Element	Al	Ca	Fe	Expected Phase
1	78.62	21.38	-	Al_4_Ca
2	79.11	20.89	-	Al_4_Ca
3	78.93	21.07	-	Al_4_Ca
4	99.68	0.32	-	(Al)
5	99.86	0.14	-	(Al)
6	91.93	2.16	5.91	(Al) + Al_4_Ca + T
7	94.82	5.05	0.13	(Al) + Al_4_Ca

**Table 2:** Tensile properties of Al-Ca alloy and Al-Ca-Fe alloy.

	Al-Ca (as-Cast)	Al-Ca (as-Rolled)	Al-Ca-Fe (as-Cast)	Al-Ca-Fe (as-Rolled)
R_p0.2_ (Mpa)	108 ± 5	166 ± 8	110 ± 4	181 ± 5
R_m_ (Mpa)	141 ± 8	194 ± 18	168 ± 4	221 ± 1
E%	13.3 ± 1.2	3.3 ± 1.1	7.1 ± 0.3	3.0 ± 0.7

**Table 3:** Comparison of nanoindentation data of different phases.

Phase	E (Gpa)	H (Gpa)	H/E	H^3^/E^2^ (Gpa)
(Al) matrix	70.2	0.443	6.31 × 10^−3^	1.76 × 10^−5^
Al_4_Ca	63.0	0.650	1.03 × 10^−2^	6.93 × 10^−5^
Al_4_Ca + T	66.2	1.25	1.89 × 10^−2^	4.46 × 10^−4^

**Table 4:** Concentrations of different regions in Figure 5 of the alloy composition in the spectrum (at%).

Element	Al	Ca	Fe
1	79.50	7.05	13.46
2	83.36	5.36	11.28
3	82.74	5.71	11.55
4	76.77	6.22	17.01

## Data Availability

Data are contained within the article.
